# A Multi-Locus Study for Detection of *Cryptosporidium* Species Isolated from Calves Population, Liverpool; UK

**Published:** 2014

**Authors:** Salman Ghaffari, Narges Kalantari, Charles A Hart

**Affiliations:** 1 *Parasitology and Mycology Group, Faculty of Medicine, Babol University of Medical Sciences, Babol, Iran.*; 2 *Cellular and Molecular Biology Research Centre (CMBRC), Babol University of Medical Sciences, Babol, Iran.*; 3 *Laboratory Sciences Group, Faculty of Paramedical, Babol University of Medical Sciences, Babol, Iran.*; 4 *Department of Medical Microbiology and Genito-Urinary Medicine, University of Liverpool, Liverpool, UK.*

**Keywords:** *Cryptosporidium*, calves, subtype, UK

## Abstract

*Cryptosporidium* is an obligate intracellular protozoan parasite infecting a wide range of hosts. The current study investigated the genetic profile of *Cryptosporidium* species in calves in Liverpool, England. Fifty-two calve fecal samples were collected from a farm and initially screened by Auramine Phenol, modified Ziehl-Neelsen and ELISA. PCR analysis of 18S rRNA gene was carried out for the positive samples. Then, positive PCR samples were genotyped by an 18S rRNA- based PCR-RFLP, COWP - based PCR- RFLP; PCR of GP60 and HSP70 genes. Additionally, sequence analysis was carried out based on representative isolates of four loci.* Cryptosporidium* oocysts and antigens were detected in 34 out of 52 (65.4%) samples using screening techniques. Genotype analysis showed the presence of *C. hominis *and* C. parvum* in one and thirteen samples, respectively. Furthermore, subtypes of *C. hominis* Ib, *C. parvum* IIa; *C. parvum* subtype 2 were identified by GP60 and HSP70 sequences, respectively. These findings indicate the diversity of the molecular characteristics of *Cryptosporidium* species in calves’ isolates. Moreover, referring to the literature; we report two new subtypes of *C. parvum *IIa and a rare case of *C. hominis* Ib in calves population.

*Cryptosporidium* is an obligate intracellular protozoan parasite infecting a wide range of vertebrate hosts such as humans, birds and cattles (1). The genus of *Cryptosporidium* consists of several genetically distinct species which are morphologically identical. There are some diagnostic methods identifying the parasite phenotypically (2). The classification methods based on parasite phenotype have limitations to distinguish the various species and genotypes found in humans and animals. Molecular genetic techniques are suitable tools to distinguish the different species and genotypes of the parasite. These methods have shown that some parasites are very host-specific while others have a wide host range. For instance, *Cryptosporidium parvum *is the most prevalent species in cattle and also is the main cause of zoonotic cryptosporidiosis in humans. It has been also found in several hosts including lamb, sheep, goat and so on (3-4).

One of the genotyping tools which are frequently used in the molecular study of this parasite are PCR and PCR- RFLP of the 18S rRNA gene. This gene is highly polymorphic within the genus and is useful as a target for the identification and differentiation of *Cryptospori-dium* species and genotypes (5-6). Another molecular tool is PCR and PCR- RFLP of the COWP gene which is a single copy gene encoding a major constituent of the inner layer of the* Cryptosporidium* oocysts wall protein (7). Moreover, PCR and sequence analysis of the 60 kDa glycoprotein (GP60) gene has been frequently used for sub-typing of various *Cryptosporidium* isolates (8). The GP60 locus has the highest resolution as a single marker for sub-typing of *C. parvum *isolates because of the existence of nearly one-hundred GP60 sub-genotypes of *C. hominis* and *C. parvum*. But this tool does not clearly divide *C. hominis* and *C. parvum* into two separate groups (9). Furthermore, heat shock protein 70 kDa (HSP70) gene is a good target for sub-typing and multi-locus study of *Cryptosporidium* isolates. This gene has a high level of heterogeneity spread over the entire sequence of a variety of *Cryptosporidium * isolates from human and animal hosts (10).

This approach was conducted to perform a multi-locus study for detection of* Cryptosporidium* species isolated from calves population using PCR, PCR- RFLP and sequence analysis of the 18S rRNA, COWP, GP60 and HSP70 gene fragments. 

## Materials and Methods

**Sample collection and screening methods**

Fifty-two fecal samples of calves with or without diarrhoea which were collected from Liverpool, Northwest England by Professor C.A. Hart, University of Liverpool, in 2003. The samples were stored at 4ºC. The fecal samples were stained by Auramine Phenol (AP) and modified Ziehl-Neelsen (MZN) methods for *Cryptosporidium* oocysts screening according to methods previously described by Casemore et al. (1985) (11). The ProSpecT ELISA kit (Alexon-Trend, Ramsey, USA) was used for detection of *Cryptosporidium* Specific Antigen (CSA) based on the manufacturer’s instruction.

**Extraction and purification of the parasite DNA**

A pea size of sample (about 200 mg) was suspended in 500 μl of ASL buffer (a stool lysis buffer) and then vortexed for 30 seconds. Oocysts were ruptured by subjecting them to a freeze- thaw cycle of + 80ºC for 15 minutes and – 80ºC for 30 minutes. DNA was extracted from the samples using the QIAamp® DNA stool mini kit (QIAGEN Ltd., Crawley, West Sussex, UK). The DNA was further purified according to the kit instruction and stored at -20°C until it was used for PCR assays. 

**PCR analysis of the 18S rRNA, COWP, GP60 and HSP70 genes**

The existence of the *Cryptosporidium* DNA in positive fecal sample either with microscopy or ELISA was verified by amplification of 18S rRNA gene fragment which produces a product of approximately 840 bp by a nested PCR. The method was performed as previously described by Xiao et al. 1999 (12-13). All DNA extracts positive at 18S rRNA gene locus were further investigated by amplification of the COWP, GP60 and HSP70 gene fragments using primers and conditions previously described by Spano et al. (1997), Zhou et al. (2003) and Sulaiman et al.(2001), respectively (7, 14-15). The primary and secondary primers used in the nested and un-nested PCR analysis of these genes, the annealing temperatures used, and sizes of the expected PCR products are listed in Table 1.

All reactions were carried out in a Biometra thermocycler in a Techne Thermal cycler  (Techne Ltd, Cambridge, UK). Positive and negative controls were added in every PCR reaction. PCR products were analyzed in 2% agarose gel stained with ethidium bromide. All primers were synthesized by Genosys Oligonucleotides (Sigma Genosys Ltd, UK).

**RFLP analysis of the 18S rRNA and COWP genes fragments**

RFLP analyses of the 18S rRNA gene fragments were performed using *Ssp*I and *Vsp*I restriction enzymes (Roche, Germany) for species identification and genotyping of *Cryptosporidium *species (12-13). Briefly, the restriction digestion was carried out at 37°C for 80 minutes. Each reaction mixture contained 15 μl of the secondary product, 1 μl of *Ssp*I (20 U), 2.5 μl of enzyme buffer and 11.5 μl of HPLC water to make a final volume of 30 μl for species identification. The* Vsp*I restriction enzyme was used at the same concentration described for *Ssp*I. RFLP analysis of the COWP gene fragment was carried out by *Rsa*I endonuclease (Roche, Germany). The restriction digestion was performed at 37°C for 4 hours in a reaction mixture according to the manufacturer’s instruction. The digestion products were analyzed in a 2% and 3.2% agarose gel stained with ethidium bromide for 18S rRNA and COWP genes fragments, respectively. Isolates were assembled according to their RFLP patterns, and a representative of each group was selected for sequence analysis.

**DNA sequences analyzing**

The PCR products of four genes targets were directly sequenced and were rubbed out from the agarose gel and purified by MicroSpin Columns Kit according to the manufacturer’s instruction (Amersham Biosciences, Buckinghamshire, UK). The Nucleotide sequences were read by the ChromasPro programme (www.technelysium). The consensus sequences and multiple alignments of the DNA sequences were edited using a nucleotide editor program (DNASTAR version 5.06, 2003). Nucleotide sequences obtained from various *Cryptosporidium* isolates were aligned with published sequences from GenBank by using National Center for Biotechnology Information (NCBI-BLAST program) (http://blast.ncbi.nlm.-nih.gov/Blast.cgi).

**The phylogeny of the GP60 gene**

The phylogenetic relationships between the GP60 sequences of the *Cryptosporidium* isolates were assessed with a NJ-tree method using the phylogenetic analysis software **Phylogeny** (16). The tree was anchored by using *C. meleagridis* as the out-group as this species showed less similarity to the other species.

**Nucleotide sequences accession numbers**

Nine sequence PCR samples used in this study have been deposited in the GenBank database under accession no: JX547009,KF533078-79, KF537685-89 and KF577776. 

## Results

**Screening Methods**

The prevalence rate of *Cryptosporidium* infection was obtained 65.4% (34/52) by screening methods. 51.2% (27/52) of the samples were positive by the AP staining method. Twenty five (48.1%) and sixteen (30.8%) out of fifty two sam-ples were positive by modified MZN and the Pro-SpecT ELISA techniques, respectively (Table 2).

**Molecular analysis of 18S rRNA gene**

**Fig 1:**
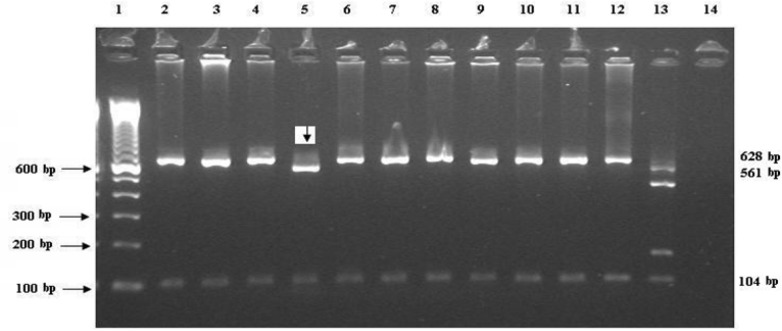
Shows the RFLP for the 18S rRNA gene by restriction endonuclease digestion patterns with *Vsp*I. Lanes are: 1, Marker (1Kb); 2-4 and 6-12, *C. Parvum *(628&104 bp bands); 5, C*. hominis *(561&104 bp bands); 13, Positive control; and 14, Negative control. The samples are from the calf collection, Liverpool, UK

The 18S rRNA gene fragment was amplified in 14 out of 34 positive samples (Table 2). *C. parvum* was identified in thirteen DNA samples and one isolate (J6) was *C. hominis *(Figure 1). Five out of fourteen DNA samples were sequenced. Sequence analysis revealed that the *C. hominis* identified by PCR - RFLP of the 18S rRNA gene had 100% homology to published sequence of *C. hominis* (accession no. AF481962). Interestingly, one isolate (3H2) which was identified as* C. parvum* using PCR- RFLP method, showed 100% sequence identity to the published strain of *C. hominis* with the above accession number. Three other sequenced isolates (17D2, D24 & Calf 44) were 99% -100% similar to the published sequence of *C. parvum* strain (accession number AY204238).

**Table 1:** Primers used in the multilocus sequence typing, nature of genetic diversity, and expected sizes of the PCR products

Locus	Primers	Sequences (5' to 3')	Fragment sites(bp)^a^	Annealing Temperat-ure(°C)	References
18S rRNA	AL1687	TTC TAG AGC TAA TAC ATG CG	156-175	55	Xiao *et al*.,1999
	AL1691	CCC TAA TCC TTC GAA ACA GGA	1455-1475		
	AL3032	GGA AGG GTT GTA TTT ATT AGA TAA AG	193-218	55	
	AL1598	AAG GAG TAA GGA ACA ACC TCC A	1008-1029		
COWP	Cry- 15	GTA GAT AAT GGA AGA GAT TGT G	921-943	52	Spano *et al.*, 1997
	Cry-9	GGA CTG AAA TAC AGG CAT TAT CTT G	1445-1470		
GP60	LX001 F1	ATA GTC TCG CTG TAT TC	4-21	50	Zhou *et al*., 2003
	LX002 R1	GCA GAG GAA CCA GCA TC	906-922		
	LX003 F2	TCC GCT GTA TTC TCA GCC	9-27	50	
	LX004 R2	GAG ATA TAT CTT GGT GCG	480-497		
HSP70	F1	ACT CTA TGA AGG TAT TGA TT	922-941	55	Sulaiman *et al.,* 2001
	R1	TTA GTC GAC CTC TTC AAC AGT TGG	2074-2051		
	F2	CAG TTG CCA TCA GTA GAG	945-962	50	
	R2	CAA CAG TTG GAC CAT TAG ATC C	2060-2039		
	INT	GGA CGA GTT TGA ACA TCA A	1831-1849		

a Expected PCR product size for both *Cryptosporidium hominis* and *C. parvum*

**Table 2:** Prevalence of *Cryptosporidium* oocyst in calve fecal samples obtained by screening and molecular techniques

Methods	Positive N %	Negative N %	Total N %
AP	27 51.2	25 48.1	52 100
MZN	25 48.1	27 51.2	52 100
ELISA	16 30.8	36 79.2	52 100
PCR of 18Sr RNA	14 41.2	20 58.8	34 100
PCR of COWP	10 71.4	4 28.6	14 100
PCR of GP60	8 57.1	6 42.9	14 100
PCR of HSP70	8 57.1	6 42.9	14 100

**Fig 2:**
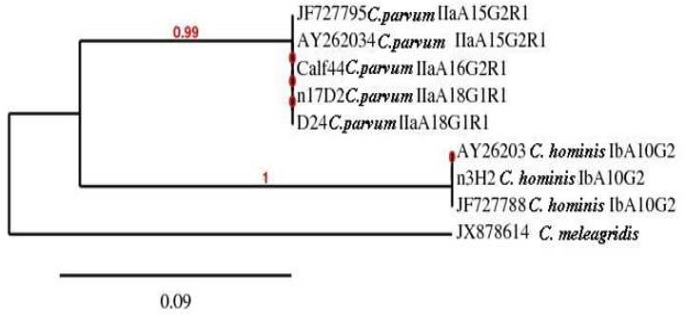
Phylogeny of *Cryptosporidium* isolates by a rooted NJ-tree based on GP60 gene. The numbers on branches are bootstrap values greater than 70% and the scale bar indicates an evolutionary distance of 0.09 nucleotides per position in the sequence. The reference sequences accession numbers are inserted

**Molecular analysis of COWP gene**

The successful amplification of *Cryptospo-ridium spp*. was observed for 10 out of 14 DNA samples based on COWP gene PCR amplification results (Table 2). PCR- RFLP of the COWP gene revealed seven isolates as *C. parvum*. In addition, abnormal bands pattern around 200 and 500 bp were observed for two samples (3H2 and 17 D2) and one sample (J6) failed by RFLP of this gene.

Of 5 sequences, two isolates (D24 & Calf44) showed 100% homology with *C. parvum* (accession number AF266273) and three samples including the isolates identified as *C. hominis* could not be assembled. 

**Molecular analysis and the** **phylogeny of GP60 gene**

Among fourteen samples positive for the 18S rRNA gene, 8 (57.1%) isolates yielded a PCR product for the GP60 locus (Table 2). Of the four sequences, three isolates (17D2, D24 & Calf44) were classified as *C. parvum* IIa allele group (accession no GQ983359 and JF727795). Interestingly, one isolate (3H2) exhibited *C. hominis* Ib allele group with 100% sequence identity with previously published sequence (accession no JF727788). Another isolate (J6) which was successfully amplified and sequenced for the 18S rRNA gene as *C. hominis*, did not yield any PCR product for sequencing of the GP60 loci.

Phylogenetic analysis of the GP60 gene using of the NJ-tree method showed that *C. hominis* and *C. parvum* isolates formed two different clades (Figure 2). The phylogenetic position of *C. hominis* GP60 subtype was consistent with its preliminary classification e.g., subtype Ib isolate (3H2) from the current study grouped with two published Ib sequences (accession numbers AY262031 and JF727788). Furthermore, three isolates of* C. parvum* IIa grouped with two published IIa sequences (accession numbers JF727795 and AY262034) forming monophyletic clades with maximum nodal support (pp=0.99).

**Molecular analysis of HSP70 gene**

Of 14 positive samples obtained by the 18S rRNA gene, 8 (57.1%) isolates yielded a PCR product for the HSP70 gene (Table 2). Upon sequence analysis, all (four isolates) were identified as *C. parvum* subtype 2 which had 100% similarity with the published isolate with accession number KC823128. The interesting aspect of this result was that isolate 3H2 which was identified as *C. hominis* by sequence analysis of 18S rRNA and GP60 genes; was designated as *C. parvum*.

## Discussion

There are several techniques for detection of *Cryptosporidium* infection in animal and human fecal samples. Generally, microscopy methods including MZN and AP and also immunoassay techniques are used to detect the parasite's oocysts and antigen (17-19). In the present study, three screening tests were used and the highest prevalence rate of *Cryptosporidium* infection (51.2%) was obtained with the AP staining method (Table 2). According to the published data, these techniques are fast and sensitive but are not able to distinguish *Cryptosporidium* species (20). The PCR analysis of 18S rRNA gene showed that 14 (41.2%) samples were positive. The presence of PCR inhibitors in fecal samples (21), the relatively low oocysts count in some of the samples (22), the extraction procedures (23), failure of cell lysis, nucleic acid degradation and capture of an insufficient amount of DNA (24) could be possible explanations for failure of yielding PCR products. These would be possible causes of unsuccessful amplification of PCR of other genes used in this study.

The proportion of C. parvum (92.9%) found in the current study by the PCR- RFLP of 18S rRNA gene was in agreement with those found in other studies (25, 27). For instance, in the UK, 93% of un-weaned calves shed oocysts of C. parvum* (*25*)*. In Northern Ireland and in the USA, *95%* and 85% of low age calves were infected with C. parvum (26-27). Interestingly, one (7.1%) *C. hominis* Ib subtype was found in the current study which suggested that calves may play a role in the transmission of this to humans. Based on our best knowledge, there are few records of *C. hominis* in calves and sheeps through the world and therefore the role of these animals in transmission of this species to humans is probably minimal (28). The first natural* C. hominis* infection in cattle was reported by Smith et al. in 2005 (29) and the second case was reported by Kang'ethe et al., which found that 4% of dairy-keeping households in an urban area in Kenya shed *C. hominis* oocysts (30). This speeies was also recorded in a goat and in a sheep in the UK (31).

The sequence analysis of the GP60 gene in this study showed that there are three *C. parvum* IIa allele group. Among these, one *C. parvum* IIa A16G2R1 (strain Calf44) and two *C. parvum* IIaA18G1R1 subgenotypes (strains 17D2 & D24) were identified for the first time on based on our best knowledge. These results are in agreement with other studies demonstrating that *C. parvum* IIa is a common subtype family in humans in addition to calves (3). Moreover, the isolate (3H2) identified as *C. hominis* by sequencing of 18S rRNA gene, was detected as *C. hominis* IbA10G2 subgenotype by sequencing of the GP60 locus. The close relationship between this subgenotype of *C.* *parvum* allele groups can be stated as a possible explanation for the presence of* C. hominis* in our samples (15, 32).

In our study, concordant results were obtained with 18S rRNA, COWP, GP60 and HSP70 genes for the majority of isolates; but some exceptions were demonstrated. The isolate (17D2) was identified as *C.* *parvum* by PCR- RFLP of 18S rRNA, GP60 and HSP70 sequences, but the COWP gene sequencing failed which may be due to mixed infections or unknown causes. Furthermore, the isolate (3H2) was detected as *C. parvum* by RFLP of 18S rRNA gene and sequences analysis of HSP70 gene but determined as *C. hominis* by sequencing of 18S rRNA and GP60 genes.

In conclusion, the multi-locus fragment analysis used in the present study detects polymorphisms in *Cryptosporidium *isolates in calves. This is the first record of two new subgenotypes of *C. parvum *IIa subjected to the GenBank data bases. This study reports *C. hominis* in calve samples and there is a rare possibility of transmission of this species from calves to humans. 
